# The associations among personality, alcohol-related Protective Behavioural Strategies (PBS), alcohol consumption and sexual intercourse in Irish, female college students

**DOI:** 10.1016/j.abrep.2017.08.001

**Published:** 2017-08-12

**Authors:** Sinéad Moylett, Brian M. Hughes

**Affiliations:** a School of Psychology, National University of Ireland Galway, University Road, Galway, Ireland; b Division of Population Medicine, School of Medicine, Cardiff University, 3rd Floor, Neuadd Meirionnydd, Heath Park, Cardiff CF14 4YS, UK

**Keywords:** Protective behavioural strategies, Personality, Women, Alcohol consumption, Sexual intercourse

## Abstract

**Introduction:**

The study presented one of the first examinations of the associations among personality, alcohol-related protective behavioural strategies (PBS), alcohol consumption, sexual intercourse and sex-related alcohol negative consequences in Irish, female college students (*n* = 522).

**Methods:**

A cross-sectional observational design was employed and participants completed the study online. Participants completed measures of personality, alcohol-related PBS, alcohol consumption and sexual intercourse. Hierarchical multiple regression was utilised to access the associations between such measures.

**Results:**

From the analyses, it was found that age, frequency of sexual intercourse, frequency of alcohol consumption, level of alcohol consumption and openness were all significantly related to the use of alcohol-related protective behavioural strategies, and in turn, sex-related negative consequences. However, inconsistent findings with other personality dimensions to those of previous research were noted.

**Conclusions:**

The findings of this study posited that the use of PBS has a key role to play in the levels of sexual intercourse and alcohol consumption, age and openness, and the associated negative sexual consequences in Irish, female college students.

## Highlights

• The use of alcohol-related PBS is related to reduced sex-related negative consequences in young, Irish females.
• Within this sample, openness was found to significantly predict the use of PBS.
• Frequency of sexual behaviour and alcohol consumption predicts the use of PBS.

## 1 Introduction

In the College Lifestyle and Attitudinal National Survey (CLAN survey), Hope, Dring, and Dring (2005) examined over 100,000 undergraduate students from 21 third level colleges in Ireland and reported that the total volume of alcohol consumed per head of student (based on the total sample of students) within one college year was 18.3 l of pure alcohol for males and 10.8 l for females. Martens et al. (2005) argued when examining alcohol consumption, a cause for serious concern can be the consequences of such consumption. Studies have shown that individuals who are defined as heavy episodic drinkers, compared to occasional heavy episodic drinkers or non-heavy episodic drinkers, are more likely to experience alcohol-related consequences (e.g., missing class, arguing with friends, not using protection when having sex, getting into trouble with the police, needing medical treatment for alcohol overdose, etc.; Wechsler et al., 2000, Wechsler et al., 2002). The CLAN survey (Hope et al., 2005) additionally found that because of alcohol consumption, 11.4% of Irish female students surveyed had taken part in unintentional sex, with 9.2% reporting unprotected sex and regular female binge drinkers reporting that they were less likely to use the contraceptive pill.

### 1.1 Protective Behavioural Strategies

In attempts to better understand and identify factors affecting alcohol consumption and the related consequences among university students, research examining cognitive-behavioural strategies (termed as “protective behavioural strategies”; PBS) and their possible association to various health-related behaviours (alcohol consumption, condom use, sexual behaviour, alcohol-related sex negative consequences) has begun (Martens et al., 2007, Lewis et al., 2009, Palmer et al., 2010, Patrick et al., 2011). PBS have been defined as cognitive-behavioural strategies used to limit or reduce the amount of alcohol consumption and/or minimize the related negative consequences that could occur from such (Benton et al., 2004, DeIva et al., 2004, Glassman et al., 2007, Martens et al., 2005, Sugarman and Carey, 2007). PBS include such strategies as alternating alcoholic drinks with non-alcoholic drinks, spacing drinks, setting a limit on the level of alcohol consumption, or using a designated driver (Lewis, Rees, Logan, Kaysen, & Kilmer, 2010). PBS are seen as offering considerable potential as they are designed to help individuals be safer or more responsible when drinking (Martens et al., 2005), and as such, are viewed as potential components for interventions to aid individuals in exercising more responsible drinking behaviours (Martens et al., 2005).

The consequences of high alcohol consumption can result in negative consequences for not just the individual (Martens et al., 2005), but additionally other students and the institutions that it could potentially affect (Perkins, 2002, Wechsler et al., 1995). Hope et al. (2005) reported that 74% of male Irish students and 65% of female Irish students experienced at least one of a range of alcohol-related harms/problems, such as “regretted things said or done after drinking” (62%), “felt effects of alcohol while at class/work” (50%), “missed school/work days” (44%) and “harmed studies/work” (28%). Furthermore, those who were classed as regular binge drinkers were more likely to partake in risk taking behaviours. Overall within the literature, findings show that those who utilise PBS consume less alcohol and experience fewer alcohol-related problems (Araas and Adams, 2008, Haines et al., 2006, Martens et al., 2007, Walters et al., 2007). In one of the first studies examining PBS, Martens et al. (2004) using a set of eight PBS, found that participants who employed PBS experienced fewer negative consequences when compared with those who did not, when controlling for alcohol consumption and gender. Similar results have been found by Benton et al. (2004) and DeIva et al. (2004) in their respective studies.

With other research suggesting an association between alcohol consumption and sex-related risks, such as greater risk of contracting an sexually transmitted infections (STIs), having multiple and/or casual partners, being less likely to discuss sex-related topics and partaking in unprotected sexual activity (Goldstein et al., 2007, Lewis et al., 2010), some studies have begun to examine the associations between alcohol consumption and specifically sex-related alcohol negative consequences (Lewis et al., 2009, Lewis et al., 2010). Due to the support that PBS are receiving throughout the literature, studies have begun to become more specialised in the kinds of PBS, samples, individual differences, drinking circumstances and related consequences which they examine, such as condom-related PBS (Lewis et al., 2009, Lewis et al., 2010), drinking during the 21st birthday week (Lewis et al., 2012), gender differences (Howard et al., 2007, Walters et al., 2007), drinking games and “prepartying” drinking behaviours (Martens et al., 2007); however, more needs to be done in terms of examining specifically sex-related negative consequences.

### 1.2 Gender differences

It should be noted that a large proportion of the research conducted on PBS and its relation to various behaviours has been conducted in the US (Lewis et al., 2009, Lewis et al., 2010, Martens et al., 2009, Nguyen et al., 2011). Furthermore, no study has examined drinking PBS and its relation to personality or specific outcomes (e.g., sex-related negative consequences) in females alone. Previous research has found results indicating differences between males and females in regard their overall use of PBS and in the types of PBS used. Studies have consistently found that women are more likely to utilise PBS, and use them more effectively, when compared with males (Benton et al., 2004, Benton et al., 2008, DeIva et al., 2004, Haines et al., 2006, Walters et al., 2007). Following on from these results, Walters et al. (2007) reported that females were more likely to use social PBS for limiting alcohol use and avoiding drinking and driving (e.g., ‘Make sure that you go home with a friend’).

In a qualitative study examining protective strategies among college freshmen, Howard et al. (2007) found that women placed a large amount of importance on knowing what the plan was for the night, and again were more likely to employ particular social protective strategies, e.g. “(1) always going back with the same group you began with, (2) girls depending on the guys in the group to look out for them and prevent them from getting into unsafe situations…and (3) group members monitoring the amount and frequency of alcohol consumption of other group members (p. 249)”. Research has suggested a few reasons for the differences in men and women in terms of their alcohol consumption and their use of PBS: women's physiology, in that they metabolise alcohol slower than men and therefore experience the effects of intoxication more quickly and intensely (LaBrie, Kenney, Lac, & Garcia, 2009); social pressure, in that women perceived more social pressure in terms of what is seen as acceptable why they drinks (DeMartini, Carey, Lao, & Luciano, 2011); and personality traits (Pervin & John, 1999). It has been suggested because of these gender differences in the use of PBS, that the employment of such strategies may have a greater impact in reducing sex-related alcohol negative consequences for females (Lewis et al., 2010).

### 1.3 Individual differences

In trying to better understand the individual differences between alcohol consumption and alcohol-related behavioural consequences, Kaly, Heesacker, and Frost (2002) posited that the alcohol myopia theory (AMT) was “the single most important theory for understanding the association between alcohol use and risky sexual behaviour” (p. 839). AMT suggests that alcohol has a causal effect on sexual behaviour, producing a “myopic” focus on cues (Steele and Josephs, 1990, Taylor and Leonard, 1983). It has been argued that an individual's sexual risk behaviour is decided by which idiosyncratic focus (on either positive or negative consequences) is most salient to that particular individual (Fromme, Karz, & Rivet, 1997). According to Davis, Hendershot, George, Norris, and Heiman (2007), “individual differences in a priori perceptions of the benefits and risks of unprotected sex may be predictive of each individual's personal relative salience of impelling versus inhibiting situational cues (p. 845)”; after consuming alcohol, these personally impelling or inhibiting cues can have more of a narrowed and concentrated focus.

The theory explains the behaviour as a kind of cognitive function impairment, in that the consumption of alcohol causes attention to be paid more to impelling cues rather than to inhibiting cues, and not all the information from the surrounding environment is processed (Abbey et al., 2004, Cooper, 2002, George and Stoner, 2000, Lewis et al., 2010). In a study comparing sober and intoxicated men, it was found that those who had consumed alcohol were more likely to say that they would have sex without a condom after watching a video of a couple who wanted to have sex but did not have a condom (MacDonald et al., 1995, MacDonald et al., 1996), suggesting that the intoxicated men paid more attention to the sexual arousal cues rather than the dangers of unprotected sex. AMT posited that individuals who use PBS consume less alcohol and therefore, can attend to both impelling and inhibiting cues, hence reducing their changes of experiencing negative consequences (Lewis et al., 2010). From this it can be suggested that to reduce the risky sexual behaviour, the level and frequency of alcohol consumption must be reduced first.

#### 1.3.1 Personality traits

Other researchers have chosen to examine individual differences in alcohol consumption through personality dimensions. The Big Five personality traits (agreeableness, conscientiousness, extroversion, neuroticism, openness; Costa & McCrae, 1985) have been shown as some of the most useful and recurrent when examining personality (Fiske, 1949, as cited in Funder, 1997). It is also the most extensively used model of personality within current research (Spence, Owens, & Goodyer, 2012). In particular, the five-factor model of personality has been displayed as a reliable measure across ages and cultures, displays good retest reliability in the same individual over time (McCrae et al., 2002, McCrae et al., 2011), and have been found to have differential associations with health behaviours (Morrison & Bennett, 2006). When examining personality in relation to sexual behaviour, sensation seeking, a trait that has been found to overlap with conscientiousness within the Big Five in terms of disinhibition within sensation seeking (Zuckerman, Kuhlman, Joireman, Teta, & Kraft, 1993), has been found across studies to have an association with sexual risk taking (Hoyle et al., 2000, Schmitt, 2004). When examining other personality dimensions, negative associations have been found between conscientiousness and risky sexual behaviour. While positive correlations have been noted between openness and risky sexual behaviours, and extraversion and risky sexual behaviour (Hoyle et al., 2000, Schmitt, 2004). The findings examining neuroticism and risky sexual behaviour are inconsistent, and examples of both positive and negative associations can be found (Schmitt, 2004).

Norris et al. (2009) found that sensation seeking, specifically sexual sensation seeking, and alcohol dose directly increased sexual arousal among women for their behavioural intentions to a hypothetical interaction leading to unprotected sex. Few studies have examined in depth the individual differences among alcohol-related PBS, alcohol consumption and sexual behaviour though. Martens et al. (2009), using the Ten Item Personality Inventory (TIPI; Gosling, Rentform, & Swann, 2003), did find that PBS mediated the associations among conscientiousness, alcohol use and alcohol-related problems, however their measure of personality was brief and the internal consistency for conscientiousness was low. Hagger-Johnson, Bewick, Conner, O'Connor, and Shickle (2011) found a significant association between conscientiousness and condom-use when examining if PBS affected the relationship between personality traits and condom use. Individuals who are viewed as conscientious are goal- and task-directed and are known for planning ahead (Pervin & John, 1999), hence they are more likely to utilise PBS. Both studies called for more research into the role of personality in PBS, specifically using more comprehensive personality measures and considering conscientiousness due to its later addition to the Big Five (Hagger-Johnson et al., 2011, Martens et al., 2009).

### 1.4 Objectives

This study aimed to examine the relationships among personality, alcohol-related PBS, alcohol consumption and sexual intercourse in Irish, female college students (18–25 years). A negative relationship between alcohol-related PBS and sex-related alcohol negative consequences was expected, as the number of PBS used increased the number of sex-related alcohol negative consequences would decrease. Furthermore, from examining previous research on Big Five dimensions and risky sexual behaviour, a positive relationship was hypothesised between conscientiousness and PBS, as well as a negative relationship between sex-related alcohol negative consequences and conscientiousness. That is, as individuals' score on conscientiousness increased, their PBS score would also increase and their reporting of sex-related alcohol negative consequences would decrease. Additionally, a positive relationship for openness and extraversion with sex-related negative consequences was hypothesised. Those with high scores on openness and extraversion would be found to have higher scores of PBS use (compared to individuals' low in openness and extraversion) and in turn, their sex-related alcohol consequences scores would decrease as their openness and extraversion scores increased.

## 2 Method

### 2.1 Design

A cross-sectional observational design was employed. Participants had to be of female gender and between the ages of 18 to 25 years. The study was completed online. After reading the information sheet and completing the consent form, participants were directed toward a 40-min online survey comprised of six self-reporting questionnaires to assess demographics, personality, social desirability, alcohol-related PBS, alcohol consumption and sexual intercourse, and sex-related alcohol negative consequences. After completing the last questionnaire, participants were debriefed and thanked for their participation. Full ethical approval was granted from the University Research Ethics Committee for the study.

### 2.2 Participants

Of all the participants invited to take part in the study, 749 gave consent and began participation; however, the numbers of individual questionnaires and for the final analysis vary due to missing data (see supplemental material S1 for missing data analysis). Due to the sensitive nature of some questions included in the study, participants were not required to give responses to all questions to continue the survey. The age range of the sample was 18–25 years (*n* = 749; *M* = 20.22, *SD* = 1.79). The nationalities of the sample were 92.2% Irish and 7.8% Non-Irish.

Participants were recruited through the student body of an Irish university and through convenience sampling from January to May of 2012. Psychology students received research credits for their participation and other participants were entered into a draw to win a €100 shopping voucher. An invitation to participate in the study was sent to the student body of the university through email, while Psychology students were invited to partake through the research credit system organised within the School. At the time of data collection, the student records reported 17,318 students registered within the university, 9811 of which were female. Between the different degree programmes in Psychology at the university, 582 female students were registered to partake in various Psychology modules.

### 2.3 Measures

Descriptive statistics and reliability analyses for all scales are provided in Table 1.

**Table 1:** Numbers (*n*) and descriptive statistics (*M*, *SD*) for measures and subscales.

Measure	Descriptive statistics		
	*n*	*M*	*SD*
*NEO 5-Factor Inventory-3*			
Neuroticism	742	39.12	7.73
Extraversion	737	41.32	6.00
Openness	742	42.38	6.81
Agreeableness	740	44.34	5.82
Conscientiousness	741	39.25	7.44
*Marlowe-Crowne Social Desirability Scale*	645	15.71	5.04
Attribution	675	10.14	2.96
Denial	667	5.57	3.00
*Alcohol-related PBS*	698	51.50	12.53
Limiting/stopping drinking	717	19.83	6.94
Manner of drinking	721	17.07	5.43
Serious harm reduction	726	14.55	3.15
*Alcohol consumption and sexual intercourse*			
Frequency of sexual intercourse within the past 6 months	650	6.20	3.86
Frequency of alcohol consumption during sexual intercourse	635	2.75	2.26
Level of alcohol consumption	632	1.72	0.86
*Sex-related alcohol negative consequences*	708	5.76	2.24

#### 2.3.1 Personality

The 60-item NEO Five Factor Inventory-3 (NEO-FFI-3; Costa & McCrae, 1985) was utilised to assess personality. The NEO-FFI-3 was chosen given the extensive use of the five-factor model of personality within current research as discussed above and furthermore, the body of evidence to support the reliability and validity of five factor models among younger individuals (Spence et al., 2012, McCrae and Costa, 2007). The personality inventory comprises five subscales (i.e., neuroticism, extraversion, openness, agreeableness and conscientiousness), each subscale consisting of 12 questions, and participants indicate how much they agreed or disagreed with each descriptive behavioural statement on a 5-point Likert scale (5 = Strongly Agree, 1 = Strongly Disagree).

Participant's individual score for each subscale was calculated and a score of 56 or higher for each dimension was considered high (Costa & McCrae, 1985). Through examination of the Cronbach's alpha values, all subscales were found to be highly reliable (neuroticism α = 0.84; extraversion α = 0.78; openness α = 0.80; agreeableness α = 0.76; conscientiousness α = 0.87).

#### 2.3.2 Social desirability

Social desirability was measured using the Marlowe-Crowne Social Desirability Scale (MCSDS; Crowne & Marlowe, 1960), as it is one of the most frequently employed social desirability questionnaire in both clinical and research settings (Beretvas, Meyers, & Leite, 2002). The MCSDS was included to try and alleviate some of the susceptibility to social desirability that exists with self-report questionnaires, particularly as some of the questions were of a sensitive nature and open to being influenced by individual's wanting to fit in with “social norms” (Crowne & Marlowe, 1960). The questionnaire contains two subscales, attribution containing 18 items (α = 0.63) and denial containing 15 items (α = 0.70), where participants are asked to indicate how true or false each statement, concerning personal attitudes and traits, was for them.

#### 2.3.3 Alcohol-related PBS

The alcohol-related PBS scale used was an updated version of the Protective Behavioural Strategies Scale (PBSS; Martens, Pederson et al., 2007; Appendix A). Following additional psychometric testing, the PBSS was updated by the original authors to increase its reliability and validity among college student drinkers (Martens, Pederson et al., 2007). The questionnaire consists of 15 items measuring various strategies used to be safer when consuming alcohol, i.e., limiting/stopping drinking (7 items, α = 0.78, e.g., “Determine not to exceed a set number of drinks”), manner of drinking (5 items, α = 0.79, e.g., “Avoid trying to ‘keep up’ or ‘out-drink’ others”), and serious harm reduction (3 items, α = 0.50, e.g., “Use a designated driver”). Participants indicate how often they engaged in each behaviour when drinking alcohol on a 6-point Likert scale. Reliability analysis for limiting/stopping drinking and manner of drinking were both found to be reliable, however for serious harm reduction the Cronbach's alpha value was reduced. This is in line with previous research and has been attributed to the small number of items within this subscale (Martens et al., 2005, Martens et al., 2007, Treloar et al., 2014, Walters et al., 2007).

#### 2.3.4 Alcohol consumption and sexual intercourse

Alcohol consumption and sexual intercourse were assessed using three questions from two previous studies on drinking and PBS (Lewis et al., 2009, Lewis et al., 2012; see Appendix B). The questions were designed to assess the frequency of sexual intercourse among participants within the past 6 months. Participants were asked to indicate how many times in the past 6 months they had had sexual intercourse on a 10-point Likert scale (1 = None, 10 = 10 or more times). Frequency of alcohol consumption when participants had taken part in sexual intercourse was measured by asking participants to specify on the occasions that they had had sexual intercourse, how many times they had consumed alcohol before or during the sexual encounter, using the same scale as the previous sexual frequency measure. If participants had consumed alcohol during a sexual encounter, they were then asked to indicate the level of alcohol they had consumed, using a 25-point scale (1 = 1 drink, 25 = 25 or more drinks).

#### 2.3.5 Sex-related alcohol negative consequences

Sex-related alcohol negative consequences were measured using a modified version of the sex-related negative consequences subscale of the Young Adult Alcohol Problem Screening Test (YAAPST; Lewis et al., 2012; see Appendix C). In their examination, Lewis et al. (2012) assessed the associations between 21st birthday alcohol consumption and related negative consequences utilising the PBSS and the YAAPST given the good reliability and validity displayed by both scales among university students (Hurlbut and Sher, 1992, Martens et al., 2005). For the current study, four items from the YAAPST related specifically to sexual negative consequences following alcohol consumption were included (e.g., regretted sexual situations, neglected to use birth control or protection against STIs). All four items on the questionnaire are measured using 5-point Likert scales (1 = Never, 5 = > 10 times), and the scale was found to be highly reliable (α = 0.85).

## 3 Analytic approach

A hierarchical multiple regression was employed to evaluate how much of an association existed among age, frequency of sexual intercourse, frequency and level of alcohol consumption, personality, social desirability, the use of alcohol-related PBS and sex-related alcohol negative consequences. In Step 1, age, frequency of sexual intercourse, frequency of alcohol consumption and level of alcohol consumption were entered. This was done to examine the effects of controlling for frequency and level of alcohol consumption as alcohol has been shown to be a risk factor for sexual negative consequences. In Step 2, the five personality dimensions and social desirability (stable psychological factors) were entered as both have been found to remain stable across time and situations (McCrae et al., 2011, Schmitt and Steyer, 1993, Stöber, 2001). Participant's total PBSS score was entered into Step 3. The analysis was structured so that the more stable psychological variables were entered into Step 2, and then the alcohol-related PBS (cognitive-behavioural strategies) in Step 3, allowing the analysis to examine the associations among these separate variables and sex-related alcohol negative consequences. 

## 4 Results

### 4.1 Descriptive statistics

While running descriptive analysis on the data, extreme outliers were noted in agreeableness, in the level of alcohol consumption and in sex-related negative consequences. However, no major differences were found between the means and the 5% trimmed means (agreeableness *M* = 44.34, *5% Trimmed M* = 44.55; alcohol consumption *M* = 1.72, *5% Trimmed M* = 1.63; sex. neg. consequences *M* = 5.76, *5% Trimmed M* = 5.48). Furthermore, casewise diagnostics completed during the regression analysis showed that < 5% of the data were outside the ± 2 limit for standardised residuals (Field, 2009) and therefore, no action was taken with the outliers.

As stated above, 749 participants gave consent and began the study, however the final participant number included in the regression model was 522. This was due to missing data on certain questionnaires (i.e., alcohol consumption and sexual intercourse). Little's MCAR test indicated that the data was indeed missing at random (i.e., no identifiable pattern exists to the missing data); χ^2^(10006) = 9740.737, *p* = 0.970.

### 4.2 Regression analysis

A correlation matrix of all variables was produced and examined for multicollinearity (see Table 2). Furthermore, all VIF values were below 2 and the average VIF score for all 11 predictors was close to 1. There were no Tolerance values below 0.2. In summary indicating that multicollinearity was not a concern. The data also met the assumption of independent errors (Durbin-Watson value = 1.91).

**Table 2:** Summary of Pearson's product correlations for all variables included in the regression model.

Variable	1	2	3	4	5	6	7	8	9	10	11	12
1. Age												
2. Sex frequency	0.09*											
3. Alcohol freq.^a^	0.00	0.43**										
4. Alcohol cons.^b^	− 0.08	− 0.01	0.35**									
5. Neuroticism	− 0.02	− 0.02	− 0.01	0.06								
6. Extraversion	− 0.05	− 0.01	0.05	0.07	− 0.36**							
7. Openness	0.14**	0.03	− 0.04	− 0.15**	0.14**	− 0.10**						
8. Agreeableness	0.01	− 0.04	− 0.16**	− 0.19**	− 0.24**	0.17**	0.06					
9. Conscientiousness	0.16**	0.10*	− 0.13**	− 0.15**	− 0.27**	0.17**	− 0.02	0.16**				
10. Social desirability	0.08*	0.07	− 0.10*	− 0.17**	− 0.41**	0.20**	− 0.01	0.50**	0.37**			
11. PBSS^c^	0.11**	0.02	− 0.30**	− 0.47**	− 0.09*	− 0.06*	0.14**	0.21**	0.38**	0.34**		
12. Sex. neg. cons.^d^	0.13**	− 0.03	0.25**	0.28**	0.07	0.00	0.04	− 0.13**	− 0.23**	− 0.20**	− 0.34**	

Significance level: *p < 0.05, **p < 0.01.

a Alcohol frequency.

b Alcohol consumption.

c Alcohol-related Protective Behavioural Strategies Scale.

d Sexual negative consequences.

The overall model was found to be significant, *F*_(11, 502)_ = 11.04, *p* < 0.001, *R*^2^ = 0.20, *Adj R*^2^ = 0.18, accounting for 18% of the variance explained in sex-related negative consequences. Step 1 did significantly predict part of the variance in sex-related negative consequences, *F*_*change*(4, 499)_ = 17.75, *p* < 0.001, *R*^2^ = 0.13, *Adj R*^2^ = 0.12. Age (β = 0.21, *p* < 0.001), frequency of sexual intercourse (β = − 0.07, *p* < 0.05), frequency of alcohol consumption (β = 0.18, *p* < 0.01) and level of alcohol consumption (β = 0.34, *p* < 0.01) were all significant predictors of sex-related negative consequences. Participant's sex-related negative consequences increased by 0.21 as age increased by one year. Their number of sex-related negative consequences decreased by 0.07 as frequency of sexual intercourse among participants increased by one time.

Lastly, their sex-related negative consequences increased by 0.18 and 0.34 as frequency of alcohol consumption and level of alcohol consumption increased by one time and one drink (respectively). Step 2 did predict a significant proportion of the variance, *F*_*change*(6, 493)_ = 3.95, *p* < 0.01, *R*^2^ = 0.17, *Adj R*^2^ = 0.15. Openness (β = 0.04, *p* < 0.01) was the only significant predictor out of the five personality dimensions and social desirability. Participant's sex-related negative consequences increased by 0.04 as their openness score increased by 1.

Additionally, Step 3 was found to significantly predict more of the variance in sex-related negative consequences, *F*_*change*(1, 492)_ = 20.41, *p* < 0.001, *R*^2^ = 0.20, *Adj R*^2^ = 0.18) and in turn, the use of alcohol-related PBS were a significant predictor (β = − 0.05, *p* < 0.001; see Table 3). It was found that sex-related negative consequences decreased by 0.05 as participant's use of alcohol-related PBS increased by 1 unit.

**Table 3:** Hierarchical regression model for predictors of sex-related negative consequences.

Variable	β	Tolerance	VIF	*R*^2^	Adj*R*^2^	*F* change
*Step 1*				0.13	0.12	17.75***
Age	0.21***	0.96	1.05			
Sex frequency	− 0.07*	0.76	1.31			
Alcohol frequency	0.18**	0.70	1.42			
Alcohol consumption	0.34*	0.78	1.28			
*Step 2*				0.17	0.15	3.95**
Neuroticism	− 0.01	0.70	1.43			
Extraversion	− 0.00	0.77	1.30			
Openness	0.04**	0.94	1.06			
Agreeableness	− 0.01	0.73	1.38			
Conscientiousness	− 0.02	0.71	1.41			
Social desirability	− 0.03	0.58	1.72			
*Step 3*				0.20	0.18	20.41***
Alcohol-related PBS	− 0.05***	0.67	1.49			

Total *R*^2^ = 0.20, Total Adj *R*^2^ = 0.18. Significance level: *p < 0.05, **p < 0.01, ***p < 0.001.

## 5 Discussion

In this study, alcohol-related PBS were found to be significantly associated with sex-related negative consequences. As the use of PBS increased, the rate of sex-related negative consequences decreased, lending further support to the use of PBS to help individuals be safer and/or more responsible when drinking (Martens et al., 2005), particularly in terms of associated negative sexual behaviours. Furthermore, PBS had medium, negative correlations with the level of alcohol frequency and consumption while taking part in sexual intercourse. Suggesting that as the use of PBS increased then the number of adverse sexual consequences experienced due to alcohol consumption decreased among this sample, which is in line with previous research (Walters et al., 2007). PBS are considered as part of the harm reduction models that attempt to alleviate the level of heavy drinking among individuals (Marlatt, Larimer, Bear, & Quigley, 1993).

The regression model was structured so that along with age and frequency of sexual intercourse, frequency and level of alcohol consumption could be controlled for. Our results support those of previous examinations (Lewis et al., 2010), as frequency and level of alcohol consumption while taking part in sexual intercourse were found to have significant correlations with sex-related negative consequences. Those individuals who consumed alcohol more frequently and in higher levels while partaking in sexual intercourse seemed to exercise fewer PBS and in turn, experience more sex-related negative consequences.

Age was significantly associated with sex-related negative consequences. Although it should be noted the samples used in this study were all young, female adults between the ages of 18–25 years. Using college students as an example, the need for better understanding of PBS utilisation among specialised population has been highlighted (Benton et al., 2004), as the cultural context in which alcohol consumption takes place can impact not only the frequency of alcohol consumption, but also individual's beliefs about when and where it is acceptable to drink and their ideas about what level of intoxication is appropriate. The Steering Group Report on a National Substance Misuse Strategy (Department of Health, 2012) and the CLAN survey (Hope et al., 2005) have both highlighted the considerable prevalence and implications of alcohol consumption among Irish young people, and it is seen within this study that younger individuals are less likely to employ PBS and in turn, can experience more sex-related negative consequences.

Among the personality dimensions, openness alone was found to be significantly correlated with sex-related negative consequences in this sample. Like previous results examining the Big Five with risky sexual behaviour (Hoyle et al., 2000, Schmitt, 2004), a positive association between openness and sex-related negative consequences was observed. When considering the correlations between openness and other variables within the model, a small, negative correlation between openness and level of alcohol consumption while taking part in sexual intercourse was noted. Also, a small, positive correlation between openness and the use of PBS was found for the sample. Individuals in this sample who were found to be higher in openness seemed to be more likely to employ PBS and in turn, potentially consumed less alcohol while taking part in sexual intercourse. Although openness has been noted as playing a role in alcohol consumption among young adults (Ibáñez et al., 2010), previous research investigating associations among personality and PBS had not found any notable results in terms of openness (Hagger-Johnson et al., 2011).

It should be acknowledged though that higher levels of openness could be related to participants being more willing to report experiencing sex-related alcohol negative consequences. This is highlighted from the results of the analyses on the missing data in this study (see S1), where participants who completed all three questions relating to sexual intercourse and alcohol consumption were found to have higher scores for sex-related alcohol negative consequences than those who did not complete the questions. This may suggest a greater openness among these participants to communicate their sexual behaviour rather than a difference in their use of PBS and/or experiences of sex-related alcohol negative consequences. Additionally, it should be noted that for this sample social desirability was not related to sex-related alcohol negative consequences, and did not appear to impact on participants reporting of such.

From previous investigations into personality dimensions and risky sexual behaviour, a few expected relationships were highlighted in relation to conscientiousness and extraversion, and their associations with sex-related negative consequences. However as mentioned above the only noted correlation from this analysis was openness. Although a significant correlation was not found in the overall model, by examining the summary of correlations conscientiousness was noted to have a number of significant correlations with variables included in the analyses (e.g., frequency of alcohol consumption, level of alcohol consumption while partaking in sexual intercourse, and sex-related alcohol negative consequences). This is in line with the results of previous research (Hagger-Johnson et al., 2011, Martens et al., 2009). Furthermore, it was found that conscientiousness had a moderate, positive relationship with PBS. Participants higher in conscientiousness appear more likely to employ PBS, this could explain the negative relationships between conscientiousness and frequency of alcohol consumption and level of alcohol consumption when taking part in sexual intercourse, and sex-related alcohol negative consequences. It is possible that conscientious individuals are more likely to exercise self-discipline, as well as being more goal- and task-directed and having a greater tendency to plan ahead (Pervin & John, 1999), therefore making them more likely to utilise responsible drinking behaviours.

### 5.1 Implications

There is little research examining personality, PBS and sex-related negative consequences in depth, and no study has examined these variables in exclusively women. Therefore, this study with its focus on young, Irish, female university students provides a unique contribution to the literature. The findings do provide additional support to the use of PBS for reducing levels of alcohol consumption and sex-related negative consequences. The results of this study highlight openness as a factor that needs to be considered particularly for a young, Irish, female sample. In line with such, it was also found that social desirability did not appear to hinder Irish female college students reporting of sexual intercourse, alcohol consumption and sex-related alcohol negative consequences. Within the current investigation of young, female university students, it was found that openness and the use of PBS are associated with sex-related negative consequences, as those who reported higher levels of openness were found to be more likely to use PBS, therefore consuming less alcohol when partaking in sexual intercourse, and in turn, experiencing fewer sex-related negative consequences. Future studies may consider examining a different model of personality, e.g., Zuckerman and Kuhlman's Alternative Five (Zuckerman, Kuhlman, Thornquist, & Kiers, 1991), which may shed more light on the role of openness or potentially sensation-seeking. Additionally, future investigations should assess if the findings carry over to other sex-related negative consequences (e.g., pressured or forced to have sex, pressure or forced someone to have sex), as well as other consequences with implications for the wider community (e.g., drove after drinking, got into physical fights, became rude, damaged property, arrested, arrested for drunken driving).

More research is warranted into personality dimensions and PBS among specialised populations as there is strong empirical support for the stability of personality over time and that changes in trait dimensions are modest (McCrae et al., 2002). Therefore, the question could be asked if it is possible to tailor drinking interventions for individuals depending on their scores on personality dimensions. In practical terms, the research can aid in the development of drinking interventions for young, female samples and assist with behaviour change in relation to alcohol consumption. PBS can be used as part of single-component preventative interventions but also as part of interventions aimed at reducing risky sexual behaviour, as the use of PBS has been found to be associated with lower consumption of alcohol during sexual intercourse in this study and in previous research (Lewis et al., 2010). Recent research has begun to examine the role of PBS in more specialised and specific situations (Lewis et al., 2012, Martens et al., 2007, Hagger-Johnson et al., 2011), the findings of such studies and of this research can aid in the development of more robust and concentrated interventions. Successful, tailored interventions implemented at the stage of university can lead to lasting life-changes for the individuals in their drinking behaviours and in turn, experienced consequences.

### 5.2 Limitations

The study had several limitations that should be noted. Firstly, a number of small considerations relating to the population; a large proportion of the sample were Irish which limits the confidence of these results being generalised to other cultures. Furthermore, the sample was restricted to women of 18–25 years, which limits the generalisability of results to other ages but strengthens the construct validity for this particular group. Information pertaining to race/ethnicity, sexuality and relationship status were not collected from participants. However as noted above 92.2% of the sample identified their nationality as Irish. These results should therefore not be generalised to other racial/ethnic groups. Also, the sample consisted of just students, which could raise questions regarding the socioeconomic status of the sample. Despite these limitations regarding the sample, it should be noted that the topics examined by this research have been shown to be quite topical for young, Irish, female adults (Hope et al., 2005).

Secondly the data were cross-sectional in nature, limiting the degree to which causal conclusions can be made. Thirdly, participants completed the study online. This was to aid in participant recruitment however it meant that the environment in which the individual completed their participation could not be controlled. Fourthly, the measure used to assess sexual intercourse is limited. The measure only assessed sexual intercourse; however there are other sexual activities that can have sex-related negative consequences. Additionally, the phrasing of the first and second measures of sexual frequency, and the limited responses (i.e. you may have had sex 30 times in the past six months but only consumed alcohol on 10 of those occasions) may have been misleading to participants.

## 6 Conclusions

The findings of this study supported previous evidence that the use of PBS has a significant negative relationship with the number of sex-related negative consequences experienced by Irish female college students. PBS have been shown in the research as useful strategies for reducing alcohol consumption, alcohol related problems and sexual risks associated with alcohol consumption (Araas and Adams, 2008, Haines et al., 2006, Walters et al., 2007). However, more extensive and in depth research is needed into specific drinking situations and events, as well as the differences between men and women in their drinking behaviours and related consequences. The findings in terms of personality dimensions are still not clear. This research found support for the importance of openness for this sample; however, the lack of findings in terms of other personality dimensions contrasts with the limited previous research that has been conducted on personality dimensions, PBS and sex-related negative consequences (Hagger-Johnson et al., 2011, Martens et al., 2009). Further investigation is warranted to better understand the factors affecting the various circumstances surrounding and the consequences associated with alcohol consumption and sexual behaviour in young, female individuals.

## Figures and Tables

**Table:** 

	Never	Rarely	Occasionally	Sometimes	Usually	Always
1. Use a designated driver	1	2	3	4	5	6
2. Determine not to exceed a set number of drinks	1	2	3	4	5	6
3. Alternative alcoholic and non-alcoholic drinks	1	2	3	4	5	6
4. Have a friend let you know when you have had enough to drink	1	2	3	4	5	6
5. Avoid drinking games	1	2	3	4	5	6
6. Leave the bar/party at a predetermines time	1	2	3	4	5	6
7. Make sure that you go home with a friend	1	2	3	4	5	6
8. Know where your drink has been at all times	1	2	3	4	5	6
9. Drink shots of liquor	1	2	3	4	5	6
10. Stop drinking at a predetermined time	1	2	3	4	5	6
11. Drink water while drinking alcohol	1	2	3	4	5	6
12. Put extra ice in your drink	1	2	3	4	5	6
13. Avoid mixing drink types of alcohol	1	2	3	4	5	6
14. Drink slowly, rather than gulp or chug	1	2	3	4	5	6
15. Avoid trying to “keep up” or “out-drink” drinks	1	2	3	4	5	6

**Figure:**
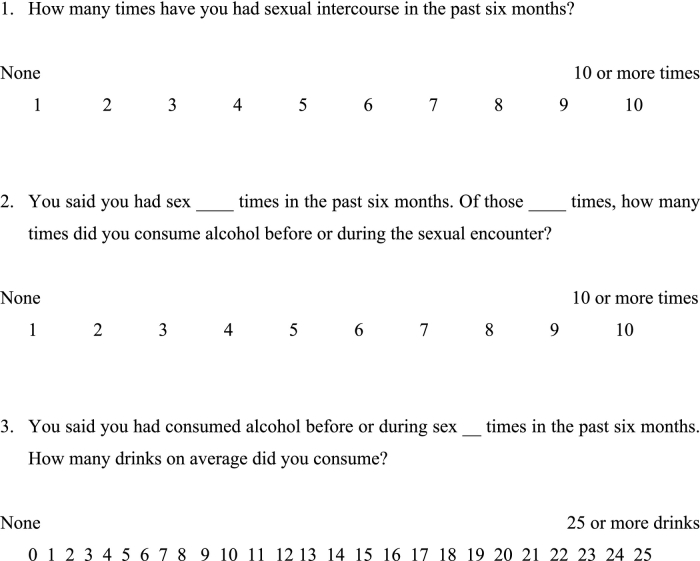


**Figure:**
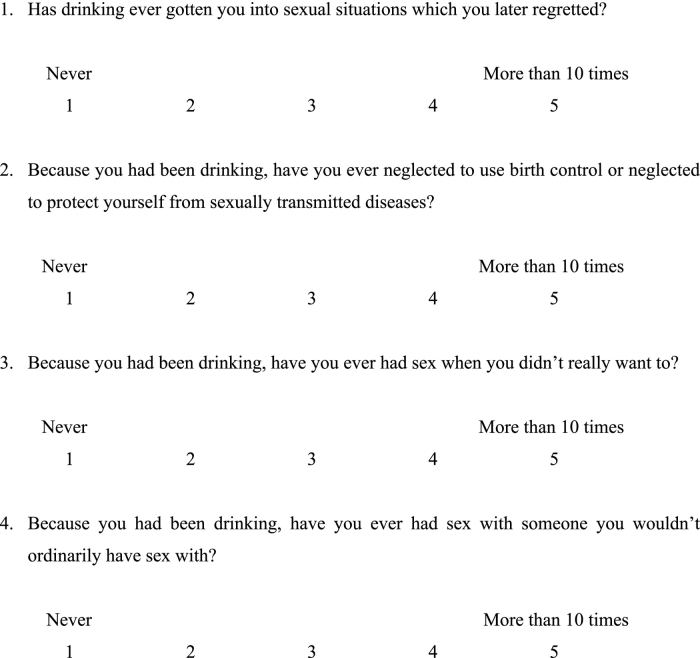


## Appendix A Alcohol-related Protective Behavioural Strategies Scale

Please indicate the degree to which you engage in the following behaviours when using alcohol.

## Appendix B Alcohol Consumption and Sexual Intercourse Scale

Please answer the following questions, where 0 equals none and 10 equals 10 or more times.

## Appendix C Sex-related Alcohol Negative Consequences Scale

Please indicate the number of times you have experienced the events below within the last six months, where 1 is equal to never and 5 is equal to > 10 times.

## Appendix D Supplementary data

Supplementary material Image 4
