# Supplementary material for: The associations among personality, alcohol-related Protective Behavioural Strategies (PBS), alcohol consumption and sexual intercourse in Irish, female college students
Source: Addict Behav Rep. 2017 Aug 12;6:56–64. doi: 10.1016/j.abrep.2017.08.001 (PMC5800550; doi:10.1016/j.abrep.2017.08.001)
Supplement: Supplementary file 1 — Supplementary material [file mmc1.docx]

**Supplemental Material 1.** Missing data analyses.

As stated in the main text, 749 participants gave consent and began the study, however the final participant number included in the regression model was 504. This was due to missing data on certain questionnaires (i.e., alcohol consumption and sexual behaviour). Little's MCAR test indicated that the data was indeed missing at random (i.e., no identifiable pattern exists to the missing data); χ^2^(11687) = 11400.13, *p* = .970.

To assess any differences between those who completed parts of the study, the following variables of interest: nationality, frequency of sexual behaviour, frequency and level of alcohol consumption while taking part in sexual behaviour, five personality dimensions, social desirability, PBSS, and sex-related negative consequences; were recoded to label the data into two groups, missing or complete. Each variable was then taken in turn and the appropriate t test or chi-square analysis completed to access any differences between the two groups (missing or complete) for that variable on all other variables. For the t tests analyses because of the multiple comparisons performed, there was a highly-inflated probability of a Type I error. Therefore, a Šidák correction was utilised, and a conservative α value of .005 was specified for the sample comparisons rather than the more usual value of .05.^47^ See below for all missing data analysis. No significant differences were noted between the missing and complete groups, bar those outlined below.

For sex-related alcohol negative consequences at the more conservative α value of .005, significant differences were noted between participants who did and did not complete the three questions relating sexual behaviour and alcohol consumption in the last 6 months: the amount of sexual behaviour they had taken part in (in the last 6 months), *U* (69, 639) = 15889.50, *p* < .005; the number of times they had consumed alcohol when they had taken part in sexual behaviour, *U* (84, 624) = 18183.50, *p* < .005; and the level of alcohol consumption when they had consumed alcohol, *U* (87, 621) = 19261.00, *p* < .005. For all three questions relating to sexual behaviour and alcohol consumption, participants who did complete the questions were found to have experienced more sex-related alcohol negative consequences than those who did not complete the questions. Furthermore, for participants’ PBS score, a significant difference was found between those who had and had not completed the question relating to the amount of alcohol consumed while taking part in sexual behaviour, *U* (92, 606) = 22708.50, *p* < .005. Those who completed the question relating to alcohol frequency were found to have used less PBS than those who did not complete the question.

**Table 1.** Comparisons between neuroticism missing and complete groups on other variables of interest

| **Variable** | **Missing Group Descriptive Statistics** | **Complete Group Descriptive Statistics** | **Test (t, U, χ^2^)** |
| --- | --- | --- | --- |
| Extraversion | *Mdn* = 43.00 (*SD* = 1.72) | *Mdn* = 42.00 (*SD* = 6.02) | *U* (6, 731) = 1791.00 |
| Openness | *M* = 38.86 (*SD* = 6.31) | *M* = 42.42 (*SD* = 6.81) | *t* (740) = -1.38 |
| Agreeableness | *M* = 45.50 (*SD* = 3.89) | *M* = 44.33 (*SD* = 5.83) | *t* (738) = 0.49 |
| Conscientiousness | *M* = 39.83 (*SD* = 4.26) | *M* = 39.24 (*SD* = 7.46) | *t* (739) = 0.19 |
| Social Desirability | *M* = 14.86 (*SD* = 6.39) | *M* = 15.72 (*SD* = 5.03) | *t* (643) = -0.45 |
| PBSS | *M* = 47.86 (*SD* = 8.92) | *M* = 51.54 (*SD* = 12.56) | *t* (696) = -0.77 |
| Sex-related alcohol negative consequences | *M* = 5.00 (*SD* = 1.29) | *M* = 5.76 (*SD* = 2.25) | *t* (706) = -0.90 |
| Sex Frequency | *Mdn* = 2 (*SD* = 3.51) | *Mdn* = 7 (*SD* = 3.86) | *U* (6, 644) = 1245.50 |
| Alcohol Frequency | *Mdn* = 1 (*SD* = 1.33) | *Mdn* = 2 (*SD* = 2.27) | *U* (6, 629) = 1445.00 |
| Alcohol Consumption | *Mdn* = 2 (*SD* = 0.75) | *Mdn* = 2 (*SD* = 0.86) | *U* (6, 626) = 1636.00 |
| Nationality | Irish = 6, Non-Irish = 1 | Irish = 681, Non-Irish = 57 | χ^2^ (1) = 0.42 |

Significance level: **p* < .05, ***p* < .01, ****p* < .001, ^ p < .005 (adjusted alpha level for multiple comparisons)

**Table 2.** Comparisons between extraversion missing and complete groups on other variables of interest

| **Variable** | **Missing Group Descriptive Statistics** | **Complete Group Descriptive Statistics** | **Test (t, U, χ^2^)** |
| --- | --- | --- | --- |
| Neuroticism | *M* = 37.45 (*SD* = 6.14) | *M* = 39.15 (*SD* = 7.75) | *t* (740) = -0.72 |
| Openness | *M* = 39.64 (*SD* = 6.33) | *M* = 42.43 (*SD* = 6.81) | *t* (740) = -1.35 |
| Agreeableness | *M* = 43.11 (*SD* = 4.70) | *M* = 44.36 (*SD* = 5.83) | *t* (738) = -0.64 |
| Conscientiousness | *M* = 37.20 (*SD* = 6.34) | *M* = 39.28 (*SD* = 7.45) | *t* (739) = -0.88 |
| Social Desirability | *M* = 15.00 (*SD* = 6.02) | *M* = 15.72 (*SD* = 5.03) | *t* (643) = -0.47 |
| PBSS | *M* = 44.91 (*SD* = 8.95) | *M* = 51.61 (*SD* = 12.56) | *t* (696) = -1.76 |
| Sex-related alcohol negative consequences | *M* = 5.83 (*SD* = 1.59) | *M* = 5.75 (*SD* = 2.25) | *t* (706) = 0.12 |
| Sex Frequency | *Mdn* = 8.50 (*SD* = 3.94) | *Mdn* = 7.00 (*SD* = 3.87) | *U* (12, 638) = 3614.50 |
| Alcohol Frequency | *Mdn* = 3.50 (*SD* = 2.71) | *Mdn* = 2.00 (*SD* = 2.25) | *U* (12, 623) = 3187.50 |
| Alcohol Consumption | *Mdn* = 2.00 (*SD* = 0.39) | *Mdn* = 2.00 (*SD* = 0.86) | *U* (12, 620) = 3999.00 |
| Nationality | Irish = 12, Non-Irish = 0 | Irish = 675, Non-Irish = 58 | χ^2^ (1) = 1.03 |

Significance level: **p* < .05, ***p* < .01, ****p* < .001, ^ p < .005 (adjusted alpha level for multiple comparisons)

**Table 3.** Comparisons between openness missing and complete groups on other variables of interest

| **Variable** | **Missing Group Descriptive Statistics** | **Complete Group Descriptive Statistics** | **Test (t, U, χ^2^)** |
| --- | --- | --- | --- |
| Neuroticism | *M* = 42.43 (*SD* = 5.86) | *M* = 39.09 (*SD* = 7.74) | *t* (740) = 1.14 |
| Extraversion | *M* = 40.00 (*SD* = 4.90) | *M* = 41.33 (*SD* = 6.01) | *t* (735) = -0.54 |
| Agreeableness | *M* = 43.83 (*SD* = 6.01) | *M* = 44.34 (*SD* = 5.82) | *t* (738) = -.21 |
| Conscientiousness | *M* = 35.29 (*SD* = 9.27) | *M* = 39.29 (*SD* = 7.41) | *t* (739) = -1.42 |
| Social Desirability | *Mdn* = 15.00 (*SD* = 2.04) | *Mdn* = 16.00 (*SD* = 5.07) | *U* (7, 638) = 1727.00 |
| PBSS | *M* = 48.67 (*SD* = 15.59) | *M* = 51.53 (*SD* = 12.51) | *t* (696) = -0.56 |
| Sex-related alcohol negative consequences | *M* = 5.71 (*SD* = 0.76) | *M* = 5.76 (*SD* = 2.25) | *t* (706) = -0.05 |
| Sex Frequency | *Mdn* = 4.50 (*SD* = 4.07) | *Mdn* = 7.00 (*SD* = 3.86) | *U* (6, 644) = 1655.50 |
| Alcohol Frequency | *Mdn* = 2.00 (*SD* = 1.63) | *Mdn* = 2.00 (*SD* = 2.27) | *U* (6, 629) = 1773.00 |
| Alcohol Consumption | *Mdn* = 2.00 (*SD* = 1.51) | *Mdn* = 2.00 (*SD* = 0.85) | *U* (6, 626) = 1431.50 |
| Nationality | Irish = 6, Non-Irish = 1 | Irish = 681, Non-Irish = 57 | χ^2^ (1) = 0.42 |

Significance level: **p* < .05, ***p* < .01, ****p* < .001, ^ p < .005 (adjusted alpha level for multiple comparisons)

**Table 4.** Comparisons between agreeableness missing and complete groups on other variables of interest

| **Variable** | **Missing Group Descriptive Statistics** | **Complete Group Descriptive Statistics** | **Test (t, U, χ^2^)** |
| --- | --- | --- | --- |
| Neuroticism | *M* = 38.38 (*SD* = 8.07) | *M* = 39.13 (*SD* = 7.73) | *t* (740) = -0.27 |
| Extraversion | *M* = 41.83 (*SD* = 6.21) | *M* = 41.32 (*SD* = 6.00) | *t* (735) = 0.21 |
| Openness | *M* = 39.63 (*SD* = 6.12) | *M* = 42.41 (*SD* = 6.81) | *t* (740) = -1.15 |
| Conscientiousness | *M* = 36.13 (*SD* = 7.86) | *M* = 39.28 (*SD* = 7.43) | *t* (739) = -1.20 |
| Social Desirability | *Mdn* = 14.00 (*SD* = 2.33) | *Mdn* = 16.00 (*SD* = 5.07) | *U* (8, 637) = 1930.50 |
| PBSS | *M* = 47.78 (*SD* = 14.57) | *M* = 51.55 (*SD* = 12.51) | *t* (696) = -0.90 |
| Sex-related alcohol negative consequences | *M* = 5.56 (*SD* = 1.94) | *M* = 5.76 (*SD* = 2.24) | *t* (706) = -0.27 |
| Sex Frequency | *Mdn* = 5.50 (*SD* = 3.76) | *Mdn* = 7.00 (*SD* = 3.87) | *U* (6, 644) = 1841.00 |
| Alcohol Frequency | *Mdn* = 2.50 (*SD* = 3.51) | *Mdn* = 2.00 (*SD* = 2.25) | *U* (6, 629) = 1770.00 |
| Alcohol Consumption | *Mdn* = 2.00 (*SD* = 1.10) | *Mdn* = 2.00 (*SD* = 0.86) | *U* (6, 626) = 1599.00 |
| Nationality | Irish = 9, Non-Irish = 0 | Irish = 678, Non-Irish = 58 | χ^2^ (1) = 0.77 |

Significance level: **p* < .05, ***p* < .01, ****p* < .001, ^ p < .005 (adjusted alpha level for multiple comparisons)

**Table 5.** Comparisons between conscientiousness missing and complete groups on other variables of interest

| **Variable** | **Missing Group Descriptive Statistics** | **Complete Group Descriptive Statistics** | **Test (t, U, χ^2^)** |
| --- | --- | --- | --- |
| Neuroticism | *M* = 39.29 (*SD* = 6.34) | *M* = 39.12 (*SD* = 7.74) | *t* (740) = 0.06 |
| Extraversion | *M* = 42.00 (*SD* = 3.63) | *M* = 41.31 (*SD* = 6.01) | *t* (735) = 0.28 |
| Openness | *M* = 41.50 (*SD* = 7.41) | *M* = 42.39 (*SD* = 6.80) | *t* (740) = -0.37 |
| Agreeableness | *M* = 43.57 (*SD* = 6.13) | *M* = 44.35 (*SD* = 5.82) | *t* (738) = -0.35 |
| Social Desirability | *Mdn* = 11.00 (*SD* = 7.64) | *Mdn* = 16.00 (*SD* = 5.02) | *U* (7, 638) = 1936.00 |
| PBSS | *M* = 51.29 (*SD* = 14.59) | *M* = 51.50 (*SD* = 12.52) | *t* (696) = -0.05 |
| Sex-related alcohol negative consequences | *M* = 5.00 (*SD* = 1.60) | *M* = 5.76 (*SD* = 2.25) | *t* (706) = -0.96 |
| Sex Frequency | *Mdn* = 10.00 (*SD* = 4.07) | *Mdn* = 7.00 (*SD* = 3.86) | *U* (8, 642) = 2163.00 |
| Alcohol Frequency | *Mdn* = 1.00 (*SD* = 1.89) | *Mdn* = 2.00 (*SD* = 2.27) | *U* (8, 627) = 2058.00 |
| Alcohol Consumption | *Mdn* = 1.00 (*SD* = 0.52) | *Mdn* = 2.00 (*SD* = 0.86) | *U* (8, 624) = 1966.50 |
| Nationality | Irish = 7, Non-Irish = 1 | Irish = 680, Non-Irish = 57 | χ^2^ (1) = 0.25 |

Significance level: **p* < .05, ***p* < .01, ****p* < .001, ^ p < .005 (adjusted alpha level for multiple comparisons)

**Table 6.** Comparisons between social desirability missing and complete groups on other variables of interest

| **Variable** | **Missing Group Descriptive Statistics** | **Complete Group Descriptive Statistics** | **Test (t, U, χ^2^)** |
| --- | --- | --- | --- |
| Neuroticism | *M* = 39.39 (*SD* = 8.17) | *M* = 39.08 (*SD* = 7.66) | *t* (740) = 0.39 |
| Extraversion | *M* = 41.12 (*SD* = 6.30) | *M* = 41.35 (*SD* = 5.95) | *t* (735) = -0.37 |
| Openness | *M* = 40.91 (*SD* = 6.89) | *M* = 42.62 (*SD* = 6.77) | *t* (740) = -2.38 |
| Agreeableness | *M* = 43.97 (*SD* = 6.38) | *M* = 44.40 (*SD* = 5.72) | *t* (738) = -0.70 |
| Conscientiousness | *M* = 40.36 (*SD* = 7.69) | *M* = 39.07 (*SD* = 7.39) | *t* (739) = 1.64 |
| PBSS | *Mdn* = 49.00 (*SD* = 16.51) | *Mdn* = 51.00 (*SD* = 11.87) | *U* (87, 611) = 25400.50 |
| Sex-related alcohol negative consequences | *M* = 5.92 (*SD* = 2.14) | *M* = 5.74 (*SD* = 2.25) | *t* (706) = 0.66 |
| Sex Frequency | *Mdn* = 10.00 (*SD* = 3.96) | *Mdn* = 7.00 (*SD* = 3.85) | *U* (68, 582) = 18665.50 |
| Alcohol Frequency | *Mdn* = 2.00 (*SD* = 2.72) | *Mdn* = 2.00 (*SD* = 2.20) | *U* (65, 570) = 17784.00 |
| Alcohol Consumption | *Mdn* = 1.00 (*SD* = 1.24) | *Mdn* = 2.00 (*SD* = 0.80) | *U* (66, 566) = 17720.50 |
| Nationality | Irish = 97, Non-Irish = 6 | Irish = 590, Non-Irish = 52 | χ^2^ (1) = 0.64 |

Significance level: **p* < .05, ***p* < .01, ****p* < .001, ^ p < .005 (adjusted alpha level for multiple comparisons)

**Table 7.** Comparisons between PBS missing and complete groups on other variables of interest

| **Variable** | **Missing Group Descriptive Statistics** | **Complete Group Descriptive Statistics** | **Test (t, U, χ^2^)** |
| --- | --- | --- | --- |
| Neuroticism | *M* = 41.06 (*SD* = 7.62) | *M* = 38.98 (*SD* = 7.72) | *t* (740) = 1.86 |
| Extraversion | *M* = 39.54 (*SD* = 5.92) | *M* = 41.45 (*SD* = 5.99) | *t* (735) = -2.18 |
| Openness | *M* = 43.02 (*SD* = 7.41) | *M* = 42.34 (*SD* = 6.76) | *t* (740) = 0.68 |
| Agreeableness | *M* = 45.25 (*SD* = 5.37) | *M* = 44.27 (*SD* = 5.85) | *t* (738) = 1.16 |
| Conscientiousness | *M* = 39.00 (*SD* = 7.98) | *M* = 39.27 (*SD* = 7.40) | *t* (739) = -0.24 |
| Social Desirability | *M* = 15.76 (*SD* = 5.56) | *M* = 15.71 (*SD* = 5.02) | *t* (643) = 0.07 |
| Sex-related alcohol negative consequences | *M* = 5.31 (*SD* = 1.90) | *M* = 5.78 (*SD* = 2.26) | *t* (706) = -1.24 |
| Sex Frequency | *Mdn* = 10.00 (*SD* = 3.86) | *Mdn* = 7.00 (*SD* = 3.87) | *U* (31, 619) = 8911.50 |
| Alcohol Frequency | *Mdn* = 2.00 (*SD* = 2.08) | *Mdn* = 2.00 (*SD* = 2.27) | *U* (29, 606) = 8340.00 |
| Alcohol Consumption | *Mdn* = 2.00 (*SD* = 0.62) | *Mdn* = 2.00 (*SD* = 0.87) | *U* (28, 604) = 8432.00 |
| Nationality | Irish = 49, Non-Irish = 2 | Irish = 638, Non-Irish = 56 | χ^2^ (1) = 1.14 |

Significance level: **p* < .05, ***p* < .01, ****p* < .001, ^ p < .005 (adjusted alpha level for multiple comparisons)

**Table 8.** Comparisons between sex-related negative consequences missing and complete groups on other variables of interest

| **Variable** | **Missing Group Descriptive Statistics** | **Complete Group Descriptive Statistics** | **Test (t, U, χ^2^)** |
| --- | --- | --- | --- |
| Neuroticism | *M* = 39.27 (*SD* = 7.42) | *M* = 39.11 (*SD* = 7.75) | *t* (740) = 0.13 |
| Extraversion | *M* = 42.02 (*SD* = 6.07) | *M* = 41.28 (*SD* = 6.00) | *t* (735) = 0.77 |
| Openness | *M* = 42.51 (*SD* = 7.05) | *M* = 42.38 (*SD* = 6.80) | *t* (740) = 0.12 |
| Agreeableness | *M* = 45.17 (*SD* = 5.01) | *M* = 44.29 (*SD* = 5.86) | *t* (738) = 0.94 |
| Conscientiousness | *M* = 42.44 (*SD* = 7.14) | *M* = 39.06 (*SD* = 7.42) | *t* (739) = 2.84 |
| Social Desirability | *M* = 16.00 (*SD* = 5.00) | *M* = 15.71 (*SD* = 5.05) | *t* (643) = 0.19 |
| PBS | *Mdn* = 56.00 (*SD* = 19.30) | *Mdn* = 51.00 (*SD* = 12.20) | *U* (26, 672) = 7536.00 |
| Sex Frequency | *Mdn* = 4.00 (*SD* = 3.75) | *Mdn* = 7.00 (*SD* = 3.86) | *U* (11, 639) = 2772.50 |
| Alcohol Frequency | *Mdn* = 1.00 (*SD* = 3.32) | *Mdn* = 2.00 (*SD* = 2.24) | *U* (11, 624) = 3175.00 |
| Alcohol Consumption | *Mdn* = 2.00 (*SD* = 0.51) | *Mdn* = 2.00 (*SD* = 0.86) | *U* (11, 621) = 3351.50 |
| Nationality | Irish = 38, Non-Irish = 2 | Irish = 649, Non-Irish = 56 | χ^2^ (1) = 0.46 |

Significance level: **p* < .05, ***p* < .01, ****p* < .001, ^ p < .005 (adjusted alpha level for multiple comparisons)

**Table 9.** Comparisons between sex frequency missing and complete groups on other variables of interest

| **Variable** | **Missing Group Descriptive Statistics** | **Complete Group Descriptive Statistics** | **Test (t, U, χ^2^)** |
| --- | --- | --- | --- |
| Neuroticism | *M* = 40.24 (*SD* = 7.36) | *M* = 38.95 (*SD* = 7.77) | *t* (740) = 1.55 |
| Extraversion | *M* = 40.92 (*SD* = 6.07) | *M* = 41.38 (*SD* = 5.99) | *t* (735) = -0.72 |
| Openness | *M* = 42.78 (*SD* = 7.02) | *M* = 42.32 (*SD* = 6.78) | *t* (740) = 0.61 |
| Agreeableness | *M* = 45.00 (*SD* = 5.36) | *M* = 44.24 (*SD* = 5.88) | *t* (738) = 1.19 |
| Conscientiousness | *M* = 40.67 (*SD* = 7.21) | *M* = 39.03 (*SD* = 7.45) | *t* (739) = 2.04 |
| Social Desirability | *M* = 15.08 (*SD* = 5.16) | *M* = 15.78 (*SD* = 5.03) | *t* (643) = -1.05 |
| PBS | *Mdn* = 54.00 (*SD* = 15.08) | *Mdn* = 50.00 (*SD* = 12.11) | *U* (79, 619) = 20695.00 |
| Sex-related alcohol negative consequences | *Mdn* = 4.00 (*SD* = 1.34) | *Mdn* = 5.00 (*SD* = 2.30) | *U* (69, 639) = 15889.50^ |
| Alcohol Frequency | *Mdn* = 2.00 (*SD* = N/A) | *Mdn* = 2.00 (*SD* = 2.26) | *U* (1, 634) = 308.00 |
| Alcohol Consumption | *Mdn* = 1 (*SD* = 0.00) | *Mdn* = 2.00 (*SD* = 0.86) | *U* (3, 629) = 435.00 |
| Nationality | Irish = 90, Non-Irish = 8 | Irish = 597, Non-Irish = 50 | χ^2^ (1) = 0.02 |

Significance level: **p* < .05, ***p* < .01, ****p* < .001, ^ p < .005 (adjusted alpha level for multiple comparisons)

**Table 10.** Comparisons between alcohol frequency missing and complete groups on other variables of interest

| **Variable** | **Missing Group Descriptive Statistics** | **Complete Group Descriptive Statistics** | **Test (t, U, χ^2^)** |
| --- | --- | --- | --- |
| Neuroticism | *M* = 39.85 (*SD* = 7.57) | *M* = 38.99 (*SD* = 7.75) | *t* (740) = 1.09 |
| Extraversion | *M* = 40.87 (*SD* = 6.19) | *M* = 41.40 (*SD* = 5.96) | *t* (735) = -0.88 |
| Openness | *M* = 42.85 (*SD* = 7.14) | *M* = 42.30 (*SD* = 6.75) | *t* (740) = 0.79 |
| Agreeableness | *M* = 45.34 (*SD* = 5.30) | *M* = 44.16 (*SD* = 5.89) | *t* (738) = 1.97 |
| Conscientiousness | *M* = 40.63 (*SD* = 7.42) | *M* = 39.00 (*SD* = 7.42) | *t* (739) = 2.17 |
| Social Desirability | *M* = 15.69 (*SD* = 5.26) | *M* = 15.71 (*SD* = 5.02) | *t* (643) = -0.03 |
| PBSS | *Mdn* = 54.00 (*SD* = 15.07) | *Mdn* = 50.00 (*SD* = 11.98) | *U* (92, 606) = 22708.50^ |
| Sex-related alcohol negative consequences | *Mdn* = 4.00 (*SD* = 1.27) | *Mdn* = 5.00 (*SD* = 2.31) | *U* (84, 624) = 18183.50^ |
| Sex Frequency | *Mdn* = 3.50 (*SD* = 2.91) | *Mdn* = 7.50 (*SD* = 3.87) | *U* (16, 634) = 3355.00 |
| Alcohol Consumption | *Mdn* = 1.00 (*SD* = 0.00) | *Mdn* = 2.00 (*SD* = 0.86) | *U* (2, 630) = 291.00 |
| Nationality | Irish = 104, Non-Irish = 9 | Irish = 583, Non-Irish = 49 | χ^2^ (1) = 0.01 |

Significance level: **p* < .05, ***p* < .01, ****p* < .001, ^ p < .005 (adjusted alpha level for multiple comparisons)

**Table 11.** Comparisons between alcohol consumption missing and complete groups on other variables of interest

| **Variable** | **Missing Group Descriptive Statistics** | **Complete Group Descriptive Statistics** | **Test (t, U, χ^2^)** |
| --- | --- | --- | --- |
| Neuroticism | *M* = 39.78 (*SD* = 7.61) | *M* = 39.00 (*SD* = 7.75) | *t* (740) = 1.01 |
| Extraversion | *M* = 40.74 (*SD* = 6.18) | *M* = 41.43 (*SD* = 5.96) | *t* (735) = -1.15 |
| Openness | *M* = 42.80 (*SD* = 7.08) | *M* = 42.31 (*SD* = 6.76) | *t* (740) = 0.72 |
| Agreeableness | *M* = 45.25 (*SD* = 5.23) | *M* = 44.17 (*SD* = 5.91) | *t* (738) = 1.83 |
| Conscientiousness | *M* = 40.80 (*SD* = 7.47) | *M* = 38.96 (*SD* = 7.40) | *t* (739) = 2.47 |
| Social Desirability | *M* = 15.38 (*SD* = 5.14) | *M* = 15.76 (*SD* = 5.03) | *t* (643) = -0.62 |
| PBSS | *Mdn* = 53.50 (*SD* = 15.05) | *Mdn* = 50.00 (*SD* = 12.00) | *U* (94, 604) = 23869.00 |
| Sex-related alcohol negative consequences | *Mdn* = 4.00 (*SD* = 1.30) | *Mdn* = 5.00 (*SD* = 2.31) | *U* (87, 621) = 19261.00^ |
| Sex Frequency | *Mdn* = 3.00 (*SD* = 3.11) | *Mdn* = 8.00 (*SD* = 3.87) | *U* (21, 629) = 4406.00 |
| Alcohol Frequency | *Mdn* = 1.00 (*SD* = 0.00) | *Mdn* = 2.00 (*SD* = 2.27) | *U* (5, 630) = 687.50 |
| Nationality | Irish = 108, Non-Irish = 8 | Irish = 579, Non-Irish = 50 | χ^2^ (1) = 0.15 |

Significance level: **p* < .05, ***p* < .01, ****p* < .001, ^ p < .005 (adjusted alpha level for multiple comparisons)
